# Effects of olympic combat sports on physical fitness in non-athlete students: a systematic review with meta-analysis

**DOI:** 10.3389/fphys.2025.1620621

**Published:** 2025-07-14

**Authors:** Jordan Hernandez-Martinez, Izham Cid-Calfucura, Eduardo Guzmán-Muñoz, Tomas Herrera-Valenzuela, Pedro Delgado-Floody, Cristian Nuñez-Espinosa, Braulio Magnani Branco, Jorge Mota, Hadi Nobari, Joaquin Perez-Carcamo, Edgar Vásquez-Carrasco, Pablo Valdés-Badilla

**Affiliations:** ^1^ Department of Physical Activity Sciences, Universidad de Los Lagos, Osorno, Chile; ^2^ Department of Education, Faculty of Humanities, Universidad de la Serena, la Serena, Chile; ^3^ Escuela de Ciencias del Deporte y Actividad Física, Facultad de Salud, Universidad Santo Tomás, UST, Santiago, Chile; ^4^ School of Kinesiology, Faculty of Health, Universidad Santo Tomás, Talca, Chile; ^5^ School of Pedagogy in Physical Education, Faculty of Education, Universidad Autónoma de Chile, Talca, Chile; ^6^ Department of Physical Activity, Sports and Health Sciences, Faculty of Medical Sciences, Universidad de Santiago de Chile (USACH), Santiago, Chile; ^7^ Department of Physical Education, Sport, and Recreation, Universidad de La Frontera, Temuco, Chile; ^8^ School of Medicine, Universidad de Magallanes, Punta Arenas, Chile; ^9^ Centro Asistencial Docente e Investigación, Universidad de Magallanes, Punta Arenas, Chile; ^10^ Interuniversity Center for Healthy Aging, Punta Arenas, Chile; ^11^ Graduate Program in Health Promotion, Cesumar University (UniCesumar), Maringá, Brazil; ^12^ Laboratory for Integrative and Translational Research in Population Health (ITR), Research Centre of Physical Activity, Health, and Leisure, Faculty of Sports, University of Porto, Porto, Portugal; ^13^ LFE Research Group, Department of Health and Human Performance, Faculty of Physical Activity and Sport Science (INEF), Universidad Politécnica de Madrid, Madrid, Spain; ^14^ School of Occupational Therapy, Faculty of Psychology, Universidad de Talca, Talca, Chile; ^15^ Centro de Investigación en Ciencias Cognitivas, Faculty of Psychology, Universidad de Talca, Talca, Chile; ^16^ Department of Physical Activity Sciences, Faculty of Education Sciences, Universidad Católica del Maule, Talca, Chile; ^17^ Sports Coach Career, Faculty of Life Sciences, Universidad Viña del Mar, Viña del Mar, Chile

**Keywords:** martial arts, physical performance, muscle strength, range of motion, adolescents OCS, olympic combat sports. VO_2_max: maximum oxygen consumption. 2.3. information and database search process

## Abstract

**Introduction:**

Olympic combat sports (OCS) present complex physical characteristics where cardiorespiratory fitness, flexibility, postural balance, endurance, agility, speed, strength, and muscular power are determinants of physical fitness. This systematic review and meta-analysis aimed to evaluate the effects of OCS interventions on selected physical fitness outcomes among school-aged and university students, compared with active or standard.

**Methods:**

A systematic literature search was conducted in May 2024 and April 2025 using seven generic databases—PubMed, ProQuest, EBSCOhost, CINAHL Complete, Scopus, Web of Science (core collection), and PEDro (Physiotherapy Evidence Database). The methodological quality and certainty of evidence were assessed using the PRISMA, TESTEX, RoB 2, and GRADE tools. The Hedge; sg effect sizes were computed. Potential sources of heterogeneity, such as subgroup analyses (type of control group, dosage training and age range), were chosen using a fixed-effects or random-effects model, with a minimum of three studies for the corresponding meta-analyses. The protocol was registered in PROSPERO (code: CRD42023391433).

**Results:**

Of 1,539 records, 9 RCTs and 4 NRCTs with 1,314 participants were included. Six overall and three subgroup meta-analyses showed significant increases in standing long jump (ES = 1.04; *p* < 0.001) and sit-and-reach (ES = 0.80; *p* < 0.05), with no significant differences (*p* > 0.05) in maximal isometric handgrip strength (MIHS; ES = 0.60), Sargent jump (ES = 0.18), VO_2_max (ES = 0.39) and 20-m shuttle run test (ES = 0.27). While in the subgroups by dosage in sit-and-reach there were significant improvements (ES = 0.90 to 1.13; *p* < 0.001) in <60 min per session and according to age range in university students in favor of OCS. Meanwhile in MIHS according to control group, there were significant increases (ES = 0.21; *p* < 0.05) in favor of OCS *versus* physical education.

**Conclusion:**

The findings suggest that OCS can be a beneficial addition in standing long jump and sit-and-reach. It does not show improvements in cardiorespiratory fitness, MIHS and Sargent jump. However, with respect to dose and age range <60 min in university students is adequate to improve sit-and-reach. OCS is more effective in improving MIHS compared to physical education.

**Systematic Review Registration:**

https://www.crd.york.ac.uk/PROSPERO/search.

## 1 Introduction

Olympic combat sports (OCS) include boxing, fencing, judo, karate, taekwondo, and wrestling (International Olympic Committee Tokyo, 2020). These present complex physical characteristics where cardiorespiratory fitness, flexibility, postural balance, endurance, agility, speed, strength, and muscular power are determinants of optimal sports performance (Cid-Calfucura et al., 2023). Various systematic reviews with meta-analyses have reported improvements in physical performance in youth (Ojeda-Aravena et al., 2023) and adults (Vasconcelos et al., 2020) OCS athletes. For example, plyometric training interventions improved muscle strength (*p* < 0.001), countermovement jump (*p* = 0.008), and agility (*p* = 0.038) in combat sport athletes (Ojeda-Aravena et al., 2023). High-intensity interval training has significantly improved (*p* = 0.0004) maximum oxygen consumption (VO_2_max) in combat sports (Vasconcelos et al., 2020).

Similarly, the OCS has positively affected metabolic, psychosocial, and anthropometric variables in the non-athlete population (Baek et al., 2021; Han and Ju, 2023; Jeong et al., 2023; Kim et al., 2021; Valdés-Badilla et al., 2021). In a systematic review conducted by Valdés-Badilla et al. (2021) in older people, it was qualitatively reported that OCS leads to a better physical-functional, physiological, and psychoemotional health status compared to control groups. A Han and Ju (2023) meta-analysis reported that taekwondo interventions in adult and older people benefit metabolic syndrome factors (*p* < 0.05) compared to active/inactive control groups. In another systematic review carried out by Kim et al. (2021), significant improvements were reported in psychosocial aspects in children and adolescent students in favor of interventions through taekwondo in behavioral subfactor labels such as behavior (*p* < 0.01), greeting (*p* < 0.01), and interpersonal (*p* = 0.01) compared to active/inactive control groups. Similarly, Baek et al. (2021), in a systematic review with meta-analysis, reported significant improvements in favor of taekwondo interventions in body fat percentage (*p* < 0.001) and muscle mass (*p* < 0.005) compared to active/inactive control groups in children, adolescents, and young adults. A systematic review with meta-analysis by Jeong et al. (2023) analyzed growth factors in Korean children and adolescent non-athletes, reporting that taekwondo training led to significantly higher levels of growth hormones (*p* < 0.001) and insulin-like growth factors (*p* < 0.001) regarding active/inactive control groups.

Physical fitness in older people and children has shown positive effects in favor of martial arts and OCS in previous systematic reviews (Stamenković et al., 2022; Valdés-Badilla et al., 2021; Valdés-Badilla et al., 2022). In a systematic review with meta-analysis carried out by Nam and Lim (2019) in Korean non-athlete students, significant improvements in physical fitness were shown in favor of taekwondo training in muscle endurance sit-up (*p* < 0.001), standing broad jump (*p* < 0.001), 20-m shuttle run test (*p* < 0.001), 200-m run (*p* < 0.001), sit-and-reach (*p* < 0.001) and postural balance with eyes closed condition (*p* < 0.001) compared to active/inactive control groups. Although there is evidence of the beneficial effect of OCS on non-athlete students (Nam and Lim, 2019), these results have been reported only in Korean students and include doctoral thesis studies. Therefore, considering that good physical fitness is associated with a better state of physical and mental health status in students (Li W. et al., 2022; Wang et al., 2023), such as the importance of presenting results from around the world in systematic reviews with meta-analysis to recommend the application of the interventions analyzed (da Costa and Jüni, 2014; Kolaski et al., 2023; Owens, 2021). Where the response to interventions through physical activity and sport may vary according to the places where they are applied depending on socio-demographic contexts (Fuentealba-Urra et al., 2022; Pharr et al., 2020), the following question is posed in the present systematic review: ¿can OCS improve health status in non-athlete students of different education levels around the world regarding to control groups performing traditional physical activity and/or carrying out their activities of daily living? This systematic review and meta-analysis aimed to evaluate the effects of OCS interventions on selected physical fitness outcomes (MIHS, Sargent jump, standing long jump, 20-m shuttle run test, VO_2_max and sit-and-reach) among school-aged and university students, compared with active or standard.

## 2 Methods

### 2.1 Protocol and registration

The Preferred Reporting Items for Systematic Reviews and Meta-analyses (PRISMA) guidelines were adhered to in this systematic review (Page et al., 2021). PROSPERO (the International Prospective Register of Systematic Reviews; ID code: CRD42023391433) has the protocol registered.

### 2.2 Eligibility criteria

The present systematic review had as inclusion criteria original peer-reviewed articles published until April 2025 that were not limited by language or publication date. Protocol records, books and book chapters, reviews, case studies, editorials, editorial letters, conference abstracts, and trials were not included. Additionally, articles were included using the PICOS (population, intervention, comparator, outcome, and study design framework) (see Table 1).

**TABLE 1:** Selection criteria used in the systematic review.

Category	Inclusion criteria	Exclusion criteria
Population (P)	Healthy students or those with a single cardiometabolic risk factor (i.e., diabetes mellitus, hypertension, dyslipidemia, overweight or obesity, among others) and/or established cardiovascular or pulmonary disease.	Non-students or students with the presence of sequelae of cardiovascular disease of the neuromuscular type (e.g., sequelae of stroke). Athletes or elite sportsmen.
Intervention (I)	Interventions with OCS (boxing, fencing, judo, karate, taekwondo, wrestling) for 4 weeks or more.	Physical activity interventions not involving OCS.
Comparator (C)	Interventions with a control group with or without supervised physical activity.	Lack of baseline and/or follow-up data. Absence of control group.
Outcome (O)	At least one physical fitness assessment by direct methods (maximal isometric handgrip strength, VO_2_max, among others) or indirect methods (the 20-m shuttle run test, sit-and-reach, standing long jump, among others).	Failure to submit a physical fitness assessment.
Study design (S)	Experimental design studies (randomized controlled and non-randomized controlled trials) with pre- and post-assessments.	Cross-sectional, retrospective, and prospective studies.

Abbreviations: OCS, Olympic combat sports; VO_2_max, maximum oxygen consumption.

### 2.3 Information and database search process

Using seven generic databases—PubMed, ProQuest, EBSCOhost, CINAHL Complete, Scopus, Web of Science (core collection), and PEDro (Physiotherapy Evidence Database), the search was carried out between May 2024 and April 2025. Free language phrases pertaining to OCS, physical fitness, students, and non-athletes were combined with Medical Subject Headings (MeSH) from the US National Library of Medicine. This was the search query that was used: (“boxing” OR “fencing” OR “judo” OR “karate” OR “taekwondo” OR “wrestling” OR “Olympic combat sports”) AND (“physical fitness” OR “physical condition” OR “physical performance” OR “performance” OR “fitness” OR “endurance” OR “strength” OR “power” OR “jump performance” OR “explosive” OR “force” OR “velocity” OR “stretch” OR “jump” OR “flexibility” OR “stretching” OR “physical exertion” OR “muscular strength” OR “muscular endurance” OR “aerobic fitness” OR “cardiorespiratory fitness” OR “cardiorespiratory capacity” OR “aerobic capacity” OR “maximum oxygen consumption” OR “VO_2_max” OR “VO_2_max” OR “VO_2_max” OR “VO_2_peak” OR “VO_2_peak” OR “VO_2_peak”) AND (“children” OR “child” OR “preschool” OR “schoolchildren” OR “young” OR “youth” OR “adolescent” OR “students” OR “young adult” OR “university students”) NOT (“athletes”). The included articles and inclusion and exclusion criteria were sent to two independent experts to help identify additional relevant studies. We established two criteria that the experts had to meet: (i) have a doctorate in sports sciences, and (ii) have peer-reviewed publications on physical fitness in different population groups and/or physical fitness in journals with an impact factor according to Journal Citation Reports®. Once all of these steps were completed, the databases were searched on 25 April 2025 to retrieve relevant errata or retractions related to the included studies. It is important to mention that the experts were not provided with our search strategy to avoid biasing their searches.

### 2.4 Studies selection and data collection process

The EndNote reference manager (version X9, Clarivate Analytics, Philadelphia, PA, United States) was used to export the studies. JHM and ICC conducted separate searches, eliminated duplicates, examined titles and abstracts, and examined complete texts. At this point, there were no disparities discovered. The procedure was carried out once again for recommendations made by outside specialists and searches inside reference lists. The entire texts of possibly suitable papers were then examined, and the rationale behind the exclusion of those that did not fit the selection criteria was disclosed. The disagreements were resolved by consensus of both authors.

### 2.5 Methodological quality assessment

This phase aimed to detect the risk of bias in each of the selected studies. For this purpose, the tool for the assessment study quality and reporting in exercise (TESTEX) scale was applied (Smart et al., 2015). This instrument is specifically designed for studies with interventions based on physical exercise. The main difference in TESTEX is that there are accommodations for assessment of whether relative exercise intensity remained constant and thus potentially prevented detraining when participants initially adapt to new exercise programs. Information on all exercise characteristics (intensity, duration, frequency, and mode) is provided to calculate exercise volume. This tool is a 15-point scale (5 points for study quality and 10 points for reporting) and addresses quality assessment criteria not listed above (eligibility criteria, randomization of participants, allocation concealed, baseline characteristics of groups, assessors blinded, outcomes of measures assessed, statistical comparison of groups, monitoring of control group, and volume and intensity of groups) specific to exercise training studies (Smart et al., 2015). Two researchers (ICC, EGM) carried out this process separately, while a third author (JHM) served as a referee for cases that were on the borderline and needed further validation from another author (PVB).

### 2.6 Data synthesis

The following data from the selected studies were obtained and analyzed: The researcher’s name and the year the work was published; the nation of origin; the study’s design; the sample’s initial health; the number of participants in the intervention and control groups; the sample’s mean age; the activities carried out in the OCS and control groups; the training volume (total duration, frequency, and time per session), the training intensity; the physical fitness data collection instruments; and the primary findings of the research. For missing data the authors communicated with the authors responsible for the study.

### 2.7 Risk of bias in individual studies

Two independent researchers (JHM and EGM) evaluated the risk of bias version 2 (RoB 2) of the included studies, and a third researcher (PVB) analyzed the results. The Cochrane Handbook for Systematic Reviews of Interventions’ principles for randomized control trials (Sterne et al., 2019) served as the foundation for this evaluation. Based on the randomization procedure, departures from the planned interventions, missing outcome data, outcome assessment, and choice of the reported result, the risk of bias was categorized as “high,” “low,” or “some concerns.”

### 2.8 Summary measures for meta-analysis

The study methodology includes meta-analysis; full information is available in PROSPERO (registration code: CRD42023391433). Meta-analyses were only performed in the present case when ≥3 studies were available (Valentine et al., 2010). Effect sizes (ES; Hedge’s g) for each physical fitness OCS and control group (CG) were calculated using the pretraining and post-training mean and standard deviation (SD) for each dependent variable. Data were standardized using the change score SD. The ES values are presented with 95% confidence intervals (95% CIs). Calculated ES was interpreted using the following scale: trivial: <0.2; small: 0.2–0.6; moderate: >0.6–1.2; large: >1.2–2.0; very large: >2.0–4.0; extremely large: >4.0 (Hopkins et al., 2009). The random-effects model was used to account for differences between studies that could affect the effect of OCS. Comprehensive Meta-analysis software (version 2.0; Biostat, Englewood, NJ, United States) was used. Statistical significance was set at p ≤ 0.05 (Verhagen et al., 1998) and was used to perform these calculations. In each trial, the random effects model (Der Simonian-Laird approach) was used to calculate and pool the standard mean deviation (SMD) and mean deviation (MD) of berg balance scale (BBS), maximal isometric handgrip strength (MIHS), Sargent jump, standing long jump, maximum oxygen consumption (VO_2_max), 20-m shuttle run test and sit-and-reach (OCS vs CG). The fundamental premise of the random effects model is that genuine effects (interventions, duration, among others) vary across studies and that samples are selected from populations with varying effect sizes. Data are pooled if at least three studies show the same (Davey et al., 2011).

Heterogeneity between trial results was tested with a Cochran’s Q test (West et al., 2010) and *I* ^2^ statistic. *I* ^2^ values of <25%, 25%–50%, and >50% represent small, medium, and large amounts of inconsistency (Higgins et al., 2003). Egger regression tests were performed to detect small study effects and possible publication bias (Higgins and Thompson, 2002). This statistic using *I* ^2^ quantifies the proportion of total variability in study effects included for analyses due to heterogeneity across studies and not due to sampling errors. After this, the model to perform the meta-analysis is selected, when heterogeneity is low, a fixed model is used, and when it is moderate to high, a random model is used.

### 2.9 Moderator analysis

Using a random effects model and an independent computed single factor analysis, potential sources of heterogeneity likely to influence training effects were selected *a priori*.

### 2.10 Subgroup analysis

Given the differences that may exist with respect to the intervention applied by the CG *versus* the experimental groups on physical performance variables (Dunn et al., 2023; Ovosi et al., 2017), we compared the type of intervention in the CG *versus* the experimental groups. Likewise, the dosage of the training, as well as the age range (Lazarus et al., 2019), were considered as possible moderating variables.

### 2.11 Certainty of evidence

Applying the Grading of Recommendations, Assessment, Development, and Evaluation (GRADE) scale (Guyatt et al., 2011) to determine the degree of certainty of evidence, the studies were classified based on four levels of evidence: high, moderate, low or very low evidence. Due to the inclusion of studies with experimental designs (randomized controlled trials and non-randomized controlled trials), all analyses began with a high degree of certainty and were downgraded if there were any doubts regarding the risk of bias, consistency, accuracy, precision, directness of results, or risk of publication bias (Guyatt et al., 2011). The studies were evaluated separately by two researchers (JHM, ICC), and any disagreements were settled by agreement with a third author (PVB).

## 3 Results

### 3.1 Study selection

**FIGURE 1:**
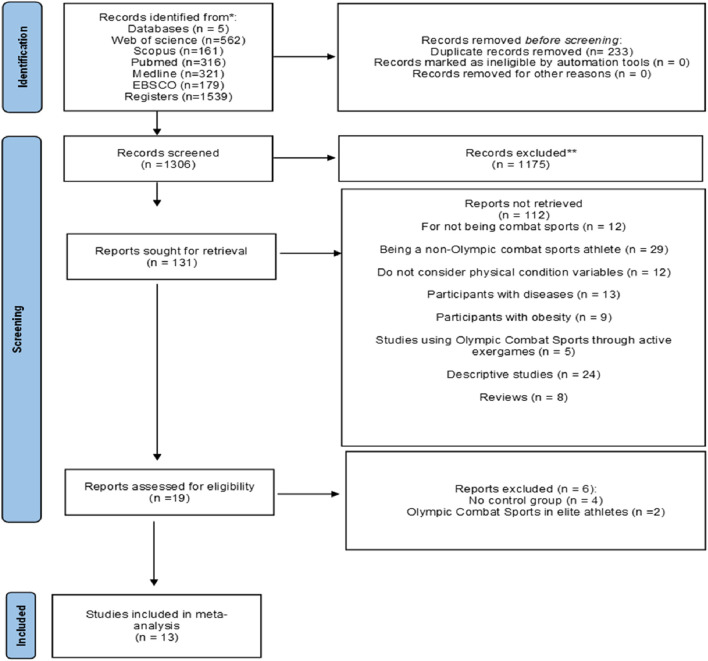
Flowchart of the review process. Legends: Based on the PRISMA guidelines(Page et al., 2021).

Figure 1 details the search process for the studies. A total of 1,539 records were found. Subsequently, duplicates were eliminated, and the studies were filtered by selecting the title, abstract, and keywords, resulting in 1,306 references. In the subsequent analysis phase, 1,175 articles were excluded because the texts did not meet the search criteria, leaving 131. Subsequently, 12 studies were for not being OCS, 29 studies in being a non-OCS athlete, 12 studies did not consider physical fitness assessments, 13 studies in participants with diseases, nine studies in participants with obesity, 5 studies using OCS through active exergames, 24 studies descriptive and 8 studies of reviews. After this process, 19 potential studies remained, of which 4 were excluded because they did not have a CG and 2 OCS in elite athletes. Where only 13 met all the selection criteria (Bae and Roh, 2021; Ju et al., 2018; Karatrantou et al., 2020; Kim et al., 2011; Mohammed, 2020; Pinto-Escalona et al., 2021; Roh et al., 2018; Roh et al., 2020; Saraiva et al., 2023; Sekulic et al., 2006; Turkeri and Ince, 2023; Violan et al., 1997; Witkowski et al., 2018).

### 3.2 Methodological quality

The 13 selected studies were analyzed using the TESTEX scale (Table 2). All studies achieved a score equal to or above 60% on the scale, namely, 12/15 (Roh et al., 2020), 11/15 (Bae and Roh, 2021; Pinto-Escalona et al., 2021; Saraiva et al., 2023; Turkeri and Ince, 2023), 10/15 (Kim et al., 2011; Mohammed, 2020), 9/15 (Roh et al., 2018) and 8/15 (Ju et al., 2018; Karatrantou et al., 2020; Sekulic et al., 2006; Violan et al., 1997; Witkowski et al., 2018), indicating moderate to high methodological quality, so no study was excluded from the systematic review.

**TABLE 2:** Study quality assessment according to the TESTEX scale.

Study	Eligibility Criteria specified	Randomly Allocated Participants	Allocation Concealed	Gorups similar at baseline	Assessors Blinded	Outcome Measures assessed >85% of participants^a^	Intention to treat analysis	Reporting of between group statistical comparisons	Point measures and measures of variability reported^b^	Activity monitoring in control group	Relative exercise Intensity reviewed	Exercise volume and energy expended	Overall TESTEX#
Kim et al. (2011)	Yes	Yes	No	Yes	No	Yes (1)	Yes	Yes	Yes (2)	No	Yes	Yes	10./15
Bae and Roh (2021)	Yes	Yes	Unclear	Yes	Unclear	Yes (1)	Yes	Yes	Yes (2)	Yes	Yes	Yes	11./15
Ju et al. (2018)	Yes	No	No	Yes	No	Yes (1)	Unclear	Yes	Yes (2)	Yes	No	Yes	8./15
Karatrantou et al. (2020)	Yes	Yes	Yes	Yes	Unclear	Yes (1)	Unclear	Yes	Unclear	No	Yes	Yes	8./15
Pinto-Escalona et al. (2021)	Yes	Yes	Yes	Unclear	Unclear	Yes (3)	Unclear	Yes	Yes (2)	Yes	Unclear	Yes	11./15
Roh et al. (2020)	Yes	Yes	Yes	Yes	Unclear	Yes (2)	Yes	Yes	Yes (2)	No	Yes	Yes	12./15
Roh et al. (2018)	Yes	Yes	Unclear	Yes	No	Yes (1)	No	Yes	Yes (2)	No	Yes	Yes	9./15
Turkeri and Ince (2023)	Yes	Yes	Yes	Yes	Yes	Yes (1)	Unclear	Yes	Yes (2)	Yes	No	Yes	11./15
Violan et al. (1997)	Yes	Unclear	Unclear	Yes	Unclear	Yes (2)	Unclear	Yes	Yes (2)	Yes	Unclear	Yes	8./15
Witkowski et al. (2018)	Yes	Yes	Unclear	Yes	Unclear	Yes (1)	Unclear	Yes	Yes (1)	Yes	No	Yes	8./15
Mohammed (2020)	Yes	Yes	No	Yes	Unclear	Yes (2)	Yes	Yes	Yes (2)	No	No	Yes	10./15
Sekulic et al. (2006)	Yes	No	No	Yes	No	Yes (1)	Unclear	Yes	Yes (2)	Yes	No	Yes	8/15
Saraiva et al. (2023)	Yes	No	No	Yes	No	Yes (2)	Unclear	Yes	Yes (2)	Yes	No	Yes	11/15

^a^ Three points are possible: one point if adherence >85%, one point if adverse events are reported, and one point if exercise attendance is reported.

^b^ Two points possible: one point if written the primary outcome is reported, one point if all different outcomes are reported. # total out of 15 points. TESTEX: Tool for assessing Study quality and reporting in Exercise (Smart et al., 2015).

### 3.3 Risk of bias within studies

**FIGURE 2:**
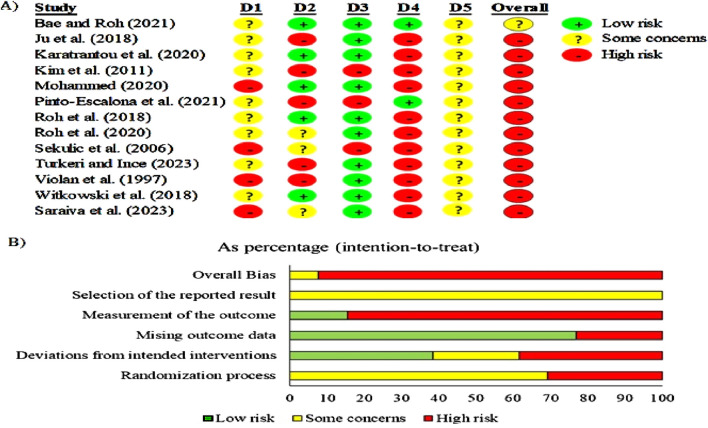
**(A)** Risk of bias within studies. Legends: D1: randomization process; D2: deviations from the intended interventions; D3: missing outcome data; D4: measurement of the outcome; D5: selection of the reported result. **(B)**. Risk of bias summary: review authors’ judgments about each risk of bias item for each included study.

The risk of bias was high for 12 studies (Ju et al., 2018; Karatrantou et al., 2020; Kim et al., 2011; Mohammed, 2020; Pinto-Escalona et al., 2021; Roh et al., 2018; Roh et al., 2020; Saraiva et al., 2023; Sekulic et al., 2006; Turkeri and Ince, 2023; Violan et al., 1997; Witkowski et al., 2018). Only one study showed some concerns (Bae and Roh, 2021). In the randomization process, nine studies showed some concerns (Bae and Roh, 2021; Ju et al., 2018; Karatrantou et al., 2020; Kim et al., 2011; Pinto-Escalona et al., 2021; Roh et al., 2018; 2020; Turkeri and Ince, 2023; Witkowski et al., 2018), and 4 showed a high risk (Mohammed, 2020; Saraiva et al., 2023; Sekulic et al., 2006; Violan et al., 1997). While in deviations from the intended interventions, 5 studies showed a low-risk (Bae and Roh, 2021; Karatrantou et al., 2020; Mohammed, 2020; Roh et al., 2018; Witkowski et al., 2018), 3 studies showed some concerns (Roh et al., 2020; Saraiva et al., 2023; Sekulic et al., 2006), and 5 showed a high-risk (Ju et al., 2018; Kim et al., 2011; Pinto-Escalona et al., 2021; Turkeri and Ince, 2023; Violan et al., 1997). In missing outcome data, 10 studies showed low-risk (Bae and Roh, 2021; Ju et al., 2018; Karatrantou et al., 2020; Mohammed, 2020; Roh et al., 2018; 2020; Saraiva et al., 2023; Turkeri and Ince, 2023; Violan et al., 1997; Witkowski et al., 2018), and 3 showed a high-risk (Kim et al., 2011; Pinto-Escalona et al., 2021; Sekulic et al., 2006). In measuring the outcome, 2 studies showed low-risk (Bae and Roh, 2021; Pinto-Escalona et al., 2021), and 11 showed high-risk (Ju et al., 2018; Karatrantou et al., 2020; Kim et al., 2011; Mohammed, 2020; Roh et al., 2018; 2020; Saraiva et al., 2023; Sekulic et al., 2006; Turkeri and Ince, 2023; Violan et al., 1997; Witkowski et al., 2018). While selecting the reported results, they all showed some concerns (Bae and Roh, 2021; Ju et al., 2018; Karatrantou et al., 2020; Kim et al., 2011; Mohammed, 2020; Pinto-Escalona et al., 2021; Roh et al., 2018; 2020; Saraiva et al., 2023; Sekulic et al., 2006; Turkeri and Ince, 2023; Violan et al., 1997; Witkowski et al., 2018). The risk of bias summary is presented in Figure 2A, and the risk of bias graph is presented in Figure 2B.

### 3.4 Studies characteristics

The variables analyzed in the 13 selected studies are listed in Table 3. Three studies in South Korea (Bae and Roh, 2021; Roh et al., 2018, Roh et al., 2020), 2 in the United States (Kim et al., 2011; Violan et al., 1997), 1 in Turkey (Turkeri and Ince, 2023), 1 in Tunez (Ju et al., 2018), 1 in Greece (Karatrantou et al., 2020), 1 in Poland (Witkowski et al., 2018), 1 in Bosnia and Herzegovina (Sekulic et al., 2006), 1 in Saudi Arabia (Mohammed, 2020), 1 in Brazil (Saraiva et al., 2023) and 1 multicenter study developed with participants from Spain, Portugal, France, Poland, and Germany (Pinto-Escalona et al., 2021). Of the 13 studies, 9 were randomized controlled trials (Bae and Roh, 2021; Ju et al., 2018; Karatrantou et al., 2020; Kim et al., 2011; Pinto-Escalona et al., 2021; Roh et al., 2018; 2020; Turkeri and Ince, 2023; Witkowski et al., 2018), while 4 were non-randomized controlled trials (Mohammed, 2020; Saraiva et al., 2023; Sekulic et al., 2006; Violan et al., 1997).

**TABLE 3:** Studies report the effects of Olympic combat sports on physical fitness in non-athlete students.

Study	Country	Study design	Sample’s initial health	Groups (n)	Mean age (years)	Type of intervention and control group	Training volume			Training intensity	Physical fitness (assessments)	Main outcomes
							Weeks	Frequency (Sessions/ week)	Session duration (minutes)			
Bae and Roh (2021)	South Korea	RCT	University students healthy	OCS: 12 CG: 12	OCS: 22.42 ± 4.40 CG: 23.25 ± 4.31	OCS: Taekwondo CG: Physical activity and recreational sports	16	1	OCS: 60 CG: No reported	50%–80% of HRmax	Maximal isometric handgrip strength Spirometry (Ebbeling protocol) Back Strength Sit-and-reach Sargent jump	**OCS *vs* CG** ↔ Maximal isometric handgrip strength ↔VO_2_max ↔Back Strength ↔Sargent jump **OCS** ↑Sit-and-reach **CG** ↔Sit-and-reach
Ju et al. (2018)	Tunez	RCT	Children and adolescents are healthy	OCS: 26 CG: 30	Both groups: 9–12	OCS: Karate CG: Physical education classes	8	2	OCS: 40 CG: 40	No reported	A target-hitting system	**OCS *vs* CG** ↔Total hit response time **OCS** ↑Total hit response time **CG** ↔Total hit response time
Karatrantou et al. (2020)	Greece	RCT	Children and adolescent are apparently healthy	OCS1: 18 OCS2: 18 CG1: 18 CG2: 18	OCS1: 9.56 ± 0.68 OCS2: 14.29 ± 1.07 CG1: 9.49 ± 0.97 CG2: 13.86 ± 1.31	OCS1: Wrestling (Greco-Roman style) OCS2 Wrestling (Greco-Roman style) CG1: Physical education classes CG2: Physical education classes	16	2	OCS: 60 CG: No reported	No reported	Maximal isometric handgrip strength	**OCS *vs* CG** ↔ Maximal isometric handgrip strength **OCS1 and OCS2** ↑ Maximal isometric handgrip strength **CG1 and CG2** ↔ Maximal isometric handgrip strength
Pinto-Escalona et al. (2021)	Multicenter (Spain, Portugal, France, Poland and Germany).	RCT	Children students apparently healthy	OCS: 388 CG: 333	OCS: 7.4 ± 0.5 CG: 7.4 ± 0.4	OCS: Karate CG: Physical education classes	36	2	OCS: 120 CG: 120	No reported	20-m shuttle run test Y-Balance test Frontal split test	**OCS *vs* CG** ↔ Cardiorespiratory fitness ↔ Balance **Both groups** ↔ Frontal split test **OCS** ↑ Cardiorespiratory fitness ↑Balance **CG** ↔ Cardiorespiratory fitness ↔Balance
Roh et al. (2020)	South Korea	RCT	Overweight or obese adolescent students	OCS: 10 CG: 10	EG: 12.60 ± 0.52 CG: 12.50 ± 0.53	OCS: Taekwondo CG: usual activities	16	5	OCS: 60 CG: no reported	No reported	Spirometry (protocol of Balke’s) Maximal isometric handgrip strength Leg strength Sit-and-reach Sargent Jump	**OCS *vs* CG** ↔VO_2_max ↔Leg strength ↔Sit-and-reach ↔Sargent jump ↔ Maximal isometric handgrip strength **Both groups** ↔ VO_2_max **OCS** ↑Leg strength ↑Sit-and-reach ↑Sargent jump ↑Handgrip strength **CG** ↔Leg strength ↔Sit and reach ↔Sargent jump ↔ Maximal isometric handgrip strength
Roh et al. (2018)	South Korea	RCT	Children students healthy	OCS: 15 CG: 15	EG: 11.53 ± 0.64 CG: 11.40 ± 0.63	OCS: Taekwondo CG: Physical education classes	16	1	OCS: 60 CG: 60	50%–80% of HR_max_	Spirometry (protocol of Nemeth) Maximal isometric handgrip strength Back Strength Sit-and-reach Sargent Jump Stork test	**OCS *vs* CG** ↔VO_2_max ↔Sit-and-reach ↔ Maximal isometric handgrip strength ↔Back Strength ↔Sargent jump ↔Stork test **Both groups** ↔VO_2_max ↔Sit-and-reach ↔Maximal isometric handgrip strength ↔Back Strength ↔Sargent jump **OCS** ↑Stork test **CG** ↔Stork test
Turkeri and Ince (2023)	Turkey	RCT	University students healthy	OCS1: 43 CG:41	OCS1: 21.2 ± 1.6 CG: 21.0 ± 1.8	OCS1: Taekwondo CG: usual activities	12	2	OCS1: 90 CG: no reported	No reported	Bass Stick Test The Plate Tapping Test	**OCS *vs* CG** ↔ Bass Stick Test ↔The Plate Tapping Test **Both groups** ↔ Bass Stick Test ↔The Plate Tapping Test
Violan et al. (1997)	United States	NRCT	Adolescent students healthy	OCS: 14 CG: 10	OCS: 10.2 ± 2.0 CG: 10.9 ± 1.4	OCS: Karate CG: Recreational sports	16	OCS: 2 CG: 3	OCS: 60 CG: 90	No reported	Static flexibility Maximal isometric handgrip strength Leg strength	**OCS *vs* CG** ↔ Maximal isometric handgrip strength ↔Leg strength ↔ Flexibility **Both groups** ↔ Maximal isometric handgrip strength ↔Leg strength ↑Flexibility
Witkowski et al. (2018)	Poland	RCT	Adolescent students healthy	OCS: 8 CG: 8	No reported	OCS: Fencing CG: usual Physical activity.	6	5	OCS: 30 CG: no reported	No reported	Maximal isometric handgrip strength	**OCS *vs* CG** ↔ Maximal isometric handgrip strength **OCS** ↑ Maximal isometric handgrip strength **CG** ↔ Maximal isometric handgrip strength
Sekulic et al. (2006)	Bosnia and Herzegovina	NRCT	Children students healthy	OCS: 41 CG: 57	OCS: 7.2 ± 0.3 CG: 7.1 ± 0.2	OCS: Judo CG: Recreational sports	36	3	OCS: 45 CG: 45	No reported	Sit-and-reach Standing long jump Sit up 20-m shuttle run test	**OCS *vs* CG** ↔Sit-and-reach ↔Standing long jump ↔Sit up ↔20-m shuttle run test **OCS** ↑Sit-and-reach ↑Standing long jump ↑Sit up ↑20-m shuttle run test **CG** ↔Sit-and-reach ↔Standing long jump ↔Sit up ↔20-m shuttle run test
Mohammed (2020)	Saudi Arabia	NRCT	University students apparently healthy	OCS1: 27 OCS2: 39 CG: 32	EG: 18–22 CG: 20–24	OCS1: Judo OCS2: Taekwondo CG: usual activities	8	2	Both EG: 50 CG: no reported	No reported	Cooper Test Curl up Sit-and-reach Standing long jump	**OCS *vs* CG** ↔Curl up ↔Sit-and-reach ↔Standing long jump ↔Cooper test **Both OCS** ↑Curl up ↑Sit-and-reach ↑Standing long jump ↔Cooper test **CG** ↔Curl up ↔Sit-and-reach ↔Standing long jump ↔Cooper test
Kim et al. (2011)	United States	RCT	Adolescent students are apparently healthy	OCS: 21 CG: 10	OCS: 15.7 ± 0.4 CG: 15.9 ± 0.6	OCS: Taekwondo CG: usual activities	12	2	OCS: 50 CG: no reported	61% of HR_max_	Spirometry (protocol of Bruce) 20-m shuttle run test 50-m shuttle run Sit-and-reach Handgrip Strength Standing long jump	**OCS *vs* CG** ↔ VO_2_max ↔20-m shuttle run test ↔50-m shuttle run ↔Sit-and-reach ↔Standing long jump **Both groups** ↔ VO_2_max ↔20-m shuttle run test ↔50-m shuttle run **OCS** ↑Sit-and-reach ↑Standing long jump **CG** ↔Sit-and-reach ↔Standing long jump
Saraiva et al. (2023)	Brazil	NRCT	Children and adolescents are healthy	OCS: 21 CG: 26	OCS: 8.95 ± 1.59 CG: 9.0 ± 2.92	OCS: Judo CG: Physical education classes	39	2	OCS: 60 CG: 60	Moderate to vigorous intensity according to the 10-point Borg scale (4–6 points).	Spirometry (one-mile run/walk test)	OCS vs CG ↔VO_2_max **OCS** ↑VO_2_max **CG** ↔VO_2_max

Abbreviations: RCT, randomized controlled trial; NRCT, non-randomized controlled trial; NR, not reported; n, number; OCS, Olympic combat sport; CG, control group; HRmax, maximum heart rate; VO_2_max, maximum oxygen consumption; ↑, significant impro vement; ↔, no significant difference.

### 3.5 Sample characteristics

Twelve studies present groups of 16–124 participants (Bae and Roh, 2021; Ju et al., 2018; Karatrantou et al., 2020; Kim et al., 2011; Mohammed, 2020; Roh et al., 2018; Roh et al., 2020; Sekulic et al., 2006; Turkeri and Ince, 2023; Violan et al., 1997; Witkowski et al., 2018), and one study presented a group of 721 participants (Pinto-Escalona et al., 2021). Consequently, the cumulative sample size across these studies amounted to 1,314 non-athlete students, composed of schoolchildren between 7 and 9 years of age (Ju et al., 2018; Karatrantou et al., 2020; Pinto-Escalona et al., 2021; Roh et al., 2018; Saraiva et al., 2023; Sekulic et al., 2006), adolescent and university students from 10 to 23 years of age (Bae and Roh, 2021; Kim et al., 2011; Mohammed, 2020; Roh et al., 2020; Saraiva et al., 2023; Turkeri and Ince, 2023; Violan et al., 1997; Witkowski et al., 2018). Five studies conducted interventions using taekwondo (Bae and Roh, 2021; Kim et al., 2011; Roh et al., 2018; Roh et al., 2020; Turkeri and Ince, 2023), 3 of karate (Ju et al., 2018; Pinto-Escalona et al., 2021; Violan et al., 1997), 3 of judo (Mohammed, 2020; Saraiva et al., 2023; Sekulic et al., 2006), 1 of fencing (Witkowski et al., 2018), and 1 of wrestling (Karatrantou et al., 2020). On the other side, 2 studies revealed that at baseline, their participants had no prior OCS experience (Bae and Roh, 2021; Kim et al., 2011), while 11 studies did not provide information on participants’ previous OCS experience (Ju et al., 2018; Karatrantou et al., 2020; Mohammed, 2020; Pinto-Escalona et al., 2021; Roh et al., 2018; Roh et al., 2020; Saraiva et al., 2023; Sekulic et al., 2006; Turkeri and Ince, 2023; Violan et al., 1997; Witkowski et al., 2018).

### 3.6 Dosing and conducted interventions

Five studies that used taekwondo (Bae and Roh, 2021; Kim et al., 2011; Roh et al., 2018; Roh et al., 2020; Turkeri and Ince, 2023), reported a duration between 8 and 16 weeks, with a frequency of 1–5 sessions per week, a time of 50–90 min, and intensity between 50% and 80% of the maximum heart rate (HR_max_) (Bae and Roh, 2021; Kim et al., 2011; Roh et al., 2018), without reporting the intensity in comparison to active CG of physical education classes (Roh et al., 2018), inactive CG (Kim et al., 2011; Mohammed, 2020; Roh et al., 2020; Turkeri and Ince, 2023), recreational sports activities (Bae and Roh, 2021), and functional groups through dance (Turkeri and Ince, 2023). Three studies (Ju et al., 2018; Pinto-Escalona et al., 2021; Violan et al., 1997) used karate as an intervention for 8–36 weeks with a frequency of 2 sessions per week between 40 min and 120 min per session, where they did not report the intensity compared to active CG of physical education classes (Ju et al., 2018; Pinto-Escalona et al., 2021) and recreational sports activities (Violan et al., 1997). Three studies (Mohammed, 2020; Saraiva et al., 2023; Sekulic et al., 2006) used judo as an intervention with a duration of 8–39 weeks with a frequency of 2–3 sessions per week between 45 min and 60 min without reporting the intensity compared to inactive CG (Mohammed, 2020) and active through recreational sports activities (Sekulic et al., 2006). One study (Witkowski et al., 2018) conducted an intervention using fencing for 6 weeks with a frequency of 5 sessions per week of 30 min without reporting the intensity compared to an inactive CG. One study (Karatrantou et al., 2020) used an intervention of wrestling for 16 weeks with a frequency of 2 sessions per week of 60 min without reporting the intensity compared to an active CG of physical education classes.

In terms of activities developed in OCS interventions, 5 studies used taekwondo (Bae and Roh, 2021; Kim et al., 2011; Roh et al., 2018; Roh et al., 2020; Turkeri and Ince, 2023), including sports techniques, such as basic stances (short step, long step, and positions), displacements with changes of direction (forward, backward, and lateral changes), punches, blocks (low, medium and high) and kicks (front, roundhouse kick, and descending). Three studies (Ju et al., 2018; Pinto-Escalona et al., 2021; Violan et al., 1997) using karate intervention that began the session consisted of non-specific motor actions to improve cardiorespiratory fitness, muscle strength, coordination, balance, and flexibility. The session was developed based on karate-specific exercises such as kicks, bipodal and unipodal jumps, and lunges with twists. The final part of the session included stretching exercises, a discussion about the class (e.g., feelings, difficulties), and final bows. Three studies (Mohammed, 2020; Saraiva et al., 2023; Sekulic et al., 2006), performed judo where the warm-up consisted of active stretching and sport-specific movements. Later, they performed various sports techniques (stances-shisei, grappling-kumi kata, movements-shintai, falls-ukemi waza, throws-nage waza, lying techniques-ne waza, and free play or sparing-randori), as well as elementary judo games, wrestling and other combat sports-related games. One study (Witkowski et al., 2018) conducted fencing intervention including eye-hand and eye-foot coordination exercises using additional equipment: fencing foil (suitable for the non-dominant hand) and an electronic fencing target for the non-dominant hand. During the last 2 weeks, the fencers practiced lunges, parries, and other fencing techniques with the non-dominant arm. They repeated the previously practiced activities on both sides at a three-to-one ratio. One study (Karatrantou et al., 2020) conducted a wrestling training program that consisted of strength exercises with medium-resistance medicine balls for the forearm, wrist, and fingers. Specifically, participants sat with their elbow flexed at 90° and squeezed the medicine ball as hard as possible at a constant pace. It is important to note that the exercise was performed with both hands at a slow to moderate speed (2 s concentric phase, 1 s isometric phase, and 3 s eccentric phase) in conjunction with wrestling techniques, such as holds, turns, and wrenches.

### 3.7 Meta-analysis results

**FIGURE 3:**
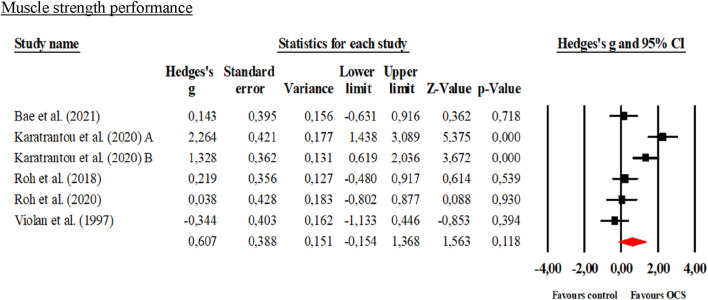
Forest plot of changes in Maximal isometric handgrip strength in students practicing Olympic combat sports compared to an active control group. Values shown correspond to effect sizes (Hedges’ g) with 95% confidence intervals (CI). The squares represent the effect sizes of each study, while the size of each square reflects the statistical weight of each study within the meta-analysis. Positive values favor students engaged in Olympic combat sports, while negative values favor the active control group.

**FIGURE 4:**
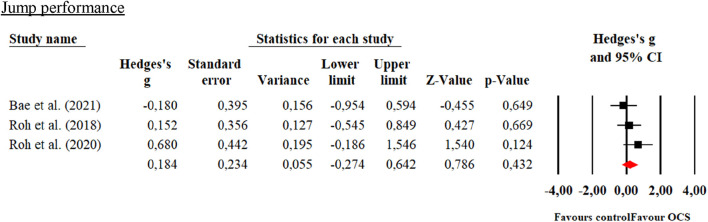
Forest plot of changes in performance on the Sargent Jump test in students practicing Olympic combat sports compared to an active control group. The values shown correspond to effect sizes (Hedges’ g) with 95% confidence intervals (CI). The squares represent the effect sizes of each study, while the size of each square reflects the statistical weight of each study within the meta-analysis. Positive values favor students practicing Olympic combat sports, while negative values favor the active control group.

**FIGURE 5:**
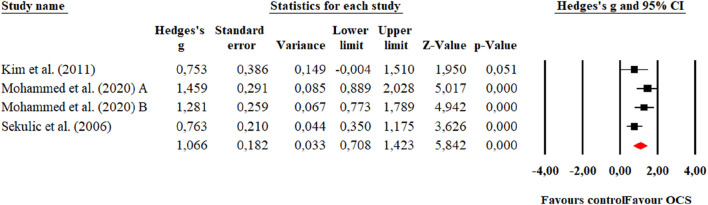
Forest plot of changes in performance on the Standing Long Jump test in students practicing Olympic combat sports compared to an active control group. The values shown correspond to effect sizes (Hedges’ g) with 95% confidence intervals (CI). The squares represent the effect sizes of each study, while the size of each square reflects the statistical weight of each study within the meta-analysis. Positive values favor students practicing Olympic combat sports, while negative values favor the active control group.

**FIGURE 6:**
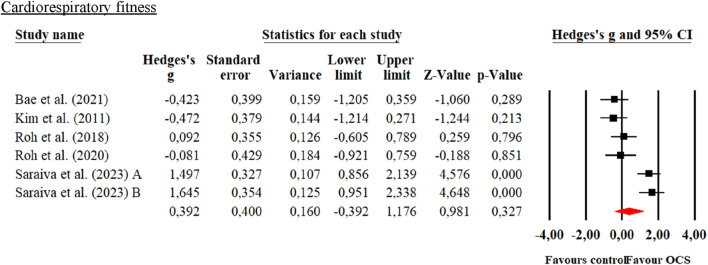
Forest plot of changes in spirometry parameters in students practicing Olympic combat sports compared with an active control group. Values shown correspond to effect sizes (Hedges’ g) with 95% confidence intervals (CI). The squares represent the effect sizes of each study, while the size of each square reflects the statistical weight of each study within the meta-analysis. Positive values favor students engaged in Olympic combat sports, while negative values favor the active control group.

**FIGURE 7:**
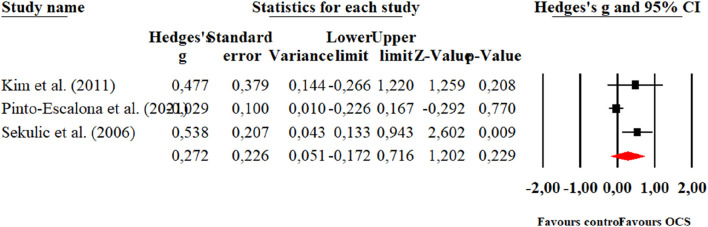
Forest plot of changes in performance on the 20-m shuttle run test in students practicing Olympic combat sports compared to an active control group. The values shown correspond to effect sizes (Hedges’ g) with 95% confidence intervals (CI). The squares represent the effect sizes of each study, while the size of each square reflects the statistical weight of each study within the meta-analysis. Positive values favor students practicing Olympic combat sports, while negative values favor the active control group.

**FIGURE 8:**
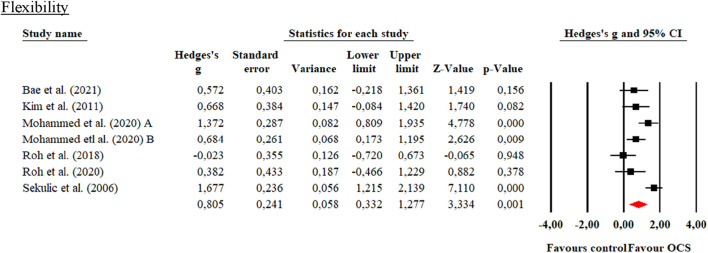
Forest plot of changes in performance on the Sit-and reach test in students practicing Olympic combat sports compared to an active control group. The values shown correspond to effect sizes (Hedges’ g) with 95% confidence intervals (CI). The squares represent the effect sizes of each study, while the size of each square reflects the statistical weight of each study within the meta-analysis. Positive values favor students practicing Olympic combat sports, while negative values favor the active control group.

The number of studies included for meta-analyses ranged from a minimum of 3–7 with a total sample size of 74–526 participants using interventions such as taekwondo, karate, judo and Greco-Roman wrestling for the experimental groups while the CG undertook physical education classes and recreational sports activities. In the global meta-analysis, when comparing OCS vs CG in the physical performance variables, only significant improvements (*p* < 0.05) were reported in favor of OCS in standing long jump and sit-and-reach with large effects (0.80–1.04). While in the MIHS, Sargent jump, VO_2_max and 20-m shuttle run test there were no significant differences (*p* > 0.05) when comparing the OCS vs CG groups with small and moderate effects (0.18–0.60). These results are presented in Table 4 and Figures 3–8.

**TABLE 4:** Effects of Olympic combat sports vs control groups on physical fitness in non-athlete students.

Physical fitness	*n* ^a^	ES (95% CI)	*p*	*I* ^2^ (%)	Egger’s test (*p*)	RW (%)
Muscle strength performance						
Maximal Isometric Handgrip Strength	6,6,6,170.	0.60 (−0.15–1.36)	0.11	82.96	0.95	1.07 to 1.14
Jump performance						
Sargent Jump	3,3,3,74.	0.18 (−0.27–0.64)	0.43	5.54	0.60	4.89 to 7.36
Standing Long Jump	4,4,4,259.	1.04 (0.70–1.42)	**0.00**	42.58	0.75	7.11 to 9.96
Cardiorespiratory fitness						
VO_2_ Max (Spirometry)	6,6,6,195.	0.39 (−0.39–1.17)	0.32	85.64	**0.10**	0.99 to 1.07.
20-m Shuttle Run Test	3,3,3, 526.	0.27 (−0.17–0.71)	0.22	71.99	0.36	4.02 to 8.70
Flexibility						
Sit-and-reach Test	7,7,7, 323.	0.80 (0.33–1.27)	**0.01**	74.30	**0.07**	2.07 to 2.85

Bolded *p*-values mean significant improvement (*p* < 0.05) in the experimental group after the Olympic combat sports intervention compared to the control group. a Data indicate the number of studies that provided data for analysis, the number of experimental groups, the number of control groups, and the total number of children, adolescents, and university students included in the analysis, respectively.

Abbreviations: 95% CI, 95% confidence interval; ES, effect sizes (Hedges’ g); RW, relative weight of each study in the analysis.

### 3.8 Meta-analysis results subgroups (physical performance)

#### 3.8.1 Maximal isometric handgrip strength

##### 3.8.1.1 Type of active control group

Regarding the type of active CG, we included analyses of OCS vs physical education and recreational activities in MIHS reporting significant improvements (*p* < 0.05) in favor of OCS vs physical education with a very large effect (1.25). No significant differences were found between OCS vs recreational activities. These results are presented in Supplementary Figure S9.

#### 3.8.2 Sit-and-Reach

##### 3.8.2.1 Age range

Regarding the age range, it was possible to compare children or adolescents and university students in sit-and-reach, presenting significant improvements (*p* < 0.05) in favor of OCS in university students with a large effect (0.90). There were no significant differences in children or adolescents. These results are presented in Supplementary Figure S10.

##### 3.8.2.2 Dosage

In terms of training dosage, we could only analyze the minutes per session (<60 min per session and ≥60 min per session) in sit-and-reach where significant improvements in favor of OCS were only reported in the <60 min session with a very long effect (1.13). No significant differences were reported in sessions to ≥60 min. These results are presented in Supplementary Figure S11. Both the results of the meta-analyses by subgroups in MIHS and sit-and-reach are presented in Table 5.

**TABLE 5:** Effects of olympic combat sports vs control groups muscle strength and flexibility in non-athlete students.

MIHS (kg)	*n* ^a^	ES (95% CI)	*p*	*I* ^2^ (%)	Egger’s test (*p*)	RW (%)
Type of active control group						
PE vs OCS*	3,3,3, 102	1.25 (0.11–2.39)	**0.03**	88.1	0.00	20.5 to 24.6
RA vs OCS	3,3,3, 68	−0.05 (−0.51 to 0.40)	0.81	**0.00**	**0.83**	2.9 to 18

**Sit-and-reach**	***n* ^a^**	**ES (95%CI)**	***p***	***I* ^2^ (%)**	**Egger’s Test (*p*)**	**RW (%)**
Age range						
Children or adolescents (CG vs OCS)	4,4,4, 133	0.70 (−0.14–1.55)	0.10	77.2	0.00	23.2 to 29.9
University Students (CG vs OCS*).	3,3,3, 154	0.90 (0.40–1.40)	**0.000**	**49**	**0.13**	33.2 to 34.9
Dosage						
<60 min per session (CG vs OCS*)	4,4,4, 213	1.13 (0.61–1.64)	**0.000**	61.7	0.04	45.4 to 50
≥60 min per session (CG vs OCS)	3,3,3, 74	0.27 (−0.16–0.72)	0.22	**0.00**	**0.54**	6.0 to 19.3

Abbreviations: *n*ª, number of total studies, number of experimental groups, number of control groups and total number of samples; *, significant statistical difference in favor of the group; CG, control group; OCS, olympic combat sport; PE, physical education; RA, recreational activities; 95% CI, 95% confidence interval; ES, effect sizes (Hedges’ g); RW, relative weight of each study in the analysis. *I* ^2^ is the heterogeneity percentage value. *p* is value significant.

### 3.9 Certainty of evidence

The results obtained in the certainty of evidence (GRADE) did not allow definitive recommendations to be made in favor of OCS as an intervention to improve physical fitness in non-athlete students because it was moderate to low (Table 6).

**TABLE 6:** GRADE assessment for the certainty of evidence.

Outcome	Study design	Risk of bias in individual studies	Risk of publication bias	Inconsistency	Indirectness	Imprecision	Certainty of evidence	Recommendation
Maximal isometric handgrip strength	4 RCT and 1 NRCT 201 participants	Moderate to High^a^	No Rated^b^	Moderate^c^	Low^d^	High^e^	Moderate to low^f^	The certainty of evidence did not allow definitive recommendations in favor of OCS as an intervention to improve maximal isometric handgrip strength.
VO_2_max	4 RCT and 1 NRCT 152 participants	Moderate to High^a^	No Rated^b^	Moderate^c^	Low^d^	High^e^	Moderate to low^f^	The certainty of evidence did not allow definitive recommendations in favor of OCS as an intervention to improve cardiorespiratory fitness.
20-m shuttle run test	2 RCT and 1 NRCT 850 participants	High^g^	No Rated^b^	Moderate^c^	Low^d^	High^e^	Low^h^	
Standing long jump	1 RCT and 2 NRCT 227 participants	High^g^	No Rated^b^	Moderate^c^	Low^d^	High^e^	Low^h^	The certainty of evidence did not allow definitive recommendations to be made in favor of OCS as an intervention to improve jump performance.
Sargent jump	3 RCT and 74 participants	Moderate to High^a^	No Rated^b^	Moderate^c^	Low^d^	High^e^	Moderate to low^f^	
Sit-and-reach	4 RCT and 2 NRCT 332 participants	Moderate to high^a^	No Rated^b^	Moderate^c^	Low^d^	High^e^	Moderate to low^f^	The certainty of evidence did not allow definitive recommendations limbs in favor of OCS as an intervention to improve lower limb flexibility.

Abbreviations: OCS, Olympic combat sports; RCT, randomized controlled trial; NRCT, non-randomized controlled trial.

^a^ Some studies have a moderate risk of bias, and others have a high risk of bias.

^b^ Not assessed due to the small number of studies.

^c^ High statistical heterogeneity (assessed through *I*2) and/or high clinical or methodological heterogeneity (interventions and study designs).

^d^ Performed. Our study performed measurements directly, so no surrogate results were used. The population (non-athletes, apparently healthy students) was clearly defined and corresponded to our objectives.

^e^ Very large 95% confidence intervals.

^f^ Moderate to high (risk of bias in individual studies), no rated (risk of publication bias), moderate (inconsistency), low (indirectness), and high (imprecision).

^g^ All studies showed a high risk.

^h^ High (risk of bias in individual studies), no rated (risk of publication bias), moderate (inconsistency), low (indirectness), and high (imprecision).

### 3.10 Adverse events and adherence

No adverse events were reported in any of the studies analyzed. All studies (Bae and Roh, 2021; Ju et al., 2018; Karatrantou et al., 2020; Kim et al., 2011; Mohammed, 2020; Pinto-Escalona et al., 2021; Roh et al., 2018; Roh et al., 2020; Saraiva et al., 2023; Sekulic et al., 2006; Turkeri and Ince, 2023; Violan et al., 1997) achieved adherence equal to or greater than 80% in taekwondo, wrestling, fencing, judo, and karate interventions. A study by Kim et al. (2011) reported non-adherence to training sessions, and 19 female adolescents dropped out due to a loss of interest in the study when they were not randomly assigned to the taekwondo group. In the study of Sekulic et al. (2006), 13 children dropped out of the training sessions due to lack of time and health problems. Also, in the study of Saraiva et al. (2023), 12 children and adolescents drop out of the training sessions due to lack of logistics.

## 4 Discussion

### 4.1 Maximal isometric handgrip strength (MIHS)

No significant improvements were found for MIHS in favor of OCS compared to active/inactive CG. Similar to what was reported by (Nam and Lim, 2019) in a meta-analysis with Korean students undergoing taekwondo training who reported no significant improvements for the dominant hand (Hedges’ g = 0.311; 95% CI = 0.005 to 0.618; p = 0.51) and non-dominant hand (Hedges’ g = 0.153; 95% CI = −0.152 to 0.458; p = 0.45) compared to active/inactive CG. This is similar to what was reported by Valdés-Badilla et al. (2023) in older people through an adapted taekwondo intervention, who reported increases in MIHS for the dominant hand of 4.0% and the non-dominant hand of 6.8%; However, these results were not statistically significant for the dominant hand (p = 0.96) and non-dominant hand (p = 0.39) compared to multicomponent training. Unlike what was reported by Roklicer et al. (2022) in national level wrestlers who reduced their body weight through wrestling training increased MIHS for the non-dominant hand (p = 0.03) compared to those who did not reduce their body weight. MIHS is a determining factor in OCS grip, especially in judo and wrestling (Muñoz-Vásquez et al., 2023). At this point, it is crucial to mention that MIHS has been shown to predict lean mass, blood pressure, physical activity levels, and upper extremity muscle strength performance (Franchini et al., 2007; Tabben et al., 2014a). In this sense, its measurement can be applied to prevent early weakness and diseases such as metabolic syndrome, diabetes, and hypertension (Tabben et al., 2014b). Our meta-analysis included five articles, only one of which reported significant improvements in MIHS (Karatrantou et al., 2020). OCS have been reported to improve MIHS in practitioners (Cid-Calfucura et al., 2023). These improvements may be mediated by specific neuromuscular adaptations resulting from OCS training. For instance, during punching actions, considerable isometric tension is generated in the hand and forearm musculature during the terminal phase of the punch. Similarly, in grappling-based disciplines such as judo and wrestling, sustained isometric contractions of the finger and wrist flexor muscles are required to counteract the opponent’s resistance, potentially promoting neuromuscular adaptations that enhance grip strength (Cid-Calfucura et al., 2023). In this sense, the high heterogeneity in our meta-analysis (*I* ^2^ = 82%) may have diluted the effects of OCS on MIHS. Specifically, the diversity in participants’ age, developmental status, sex, and health status (e.g., healthy adolescents *versus* obese adolescents) may have influenced the positive effect of OCS.

### 4.2 Cardiorespiratory fitness

VO_2_max was meta-analyzed by spirometry in cardiorespiratory fitness and showed no significant changes for or against OCS compared to active/inactive CG. These results are different from those reported by Muñoz-Vásquez et al. (2023) in a systematic review of non-athletes where significant improvements (SMD = 4.61; 95% CI = 1.46 to 7.76; *I* ^2^ = 99%; *p* = 0.004) in favor of OCS in VO_2_max by spirometry compared to active/inactive CG were presented. These results are similar to those presented by Stamenković et al. (2022) in a systematic review reported that karate, taekwondo, aikido, and judo led to improvements in VO_2_max in preschoolers and schoolchildren compared to active/inactive CG. Practicing OCS disciplines such as taekwondo and boxing resembles high-intensity interval training (HIIT) due to the intermittent nature of their explosive actions (Huerta Ojeda et al., 2017). This training modality may enhance oxygen delivery and utilization capacities, as well as improve gas exchange efficiency at both the pulmonary and muscular levels (Ojeda-Aravena et al., 2021). However, in the present meta-analysis, no significant differences in VO_2_max were reported in children, adolescents, and young students. Regarding the findings in this systematic review, first of all, it is essential to mention that VO_2_max values in combat sports athletes are not usually very high; for example, Franchini et al. (2007) in elite Brazilian judokas have reported VO_2_max values of 58.1 ± 10.8 mL.kg^−1^. min^−1^. Marković et al. (2005) reported VO_2_max values of 49.6 ± 3.3 mL.kg^−1^. min^−1^ in taekwondo in international-level athletes. In karate, Tabben et al. (2014a) found VO_2_max values of 53.7 ± 5.1 mL.kg^−1^. min^−1^ in international-level athletes. In this sense, there is the possibility that the students have already had VO_2_max values close to or within the reference values mentioned and that the practice of OCS without competitive purposes has not caused sufficient stimulation to generate physiological adaptations. About the thing before, this may be due to the training frequency of the studies analyzed and the intensity of the interventions; Kim et al. (2011) and Mohammed (2020) only applied for the training program twice a week, given the characteristics of the participants, who were underage students and had to comply with their school schedule. According to Carazo and Moncada-Jiménez (2015), a frequency of at least 3 days a week is recommended to improve VO_2_max, which is limited by the oxygen supply, where cardiac output is one of the main factors that determine its performance (Abarzúa et al., 2019). On the other hand, the training intensity was not reported in two of the studies analyzed (Kim et al., 2011; Sekulic et al., 2006), which is vital to consider to cause increases in cardiorespiratory fitness, establishing itself in an optimal range to work on 55%–90% of HR_max_ (Helgerud et al., 2007).

Another test that could be meta-analyzed for cardiorespiratory fitness was the 20-m shuttle run test, which showed no significant improvement for or against OCS compared to active/inactive CG. These results are contradictory to those reported by Muñoz-Vásquez et al. (2023) in a systematic review of OCS in a non-athlete population where significant improvements (*p* ˃ 0.05) in 20-m shuttle run test were presented in favor of OCS in comparison to CG. Similar to that reported by Ojeda-Aravena et al. (2021) in young taekwondo athletes who showed significant improvements (*p* < 0.05) in cardiorespiratory fitness by 20-m shuttle run test in a 4-week specific HIIT program regarding traditional taekwondo training. Another study conducted by Ouergui et al. (2020) in adolescent taekwondo athletes showed significant improvements (*p* < 0.001) in a 20-m shuttle run test in favor of a 4-week HIIT intervention compared to an inactive CG. This contradiction concerning the systematic review, like the findings in VO_2_max, may be related to the different frequencies, intensities, and modalities of the interventions. Specifically, regarding the meta-analyzed articles, two studies had a frequency of once a week with an intensity between 50% and 80% HR_max_ (Bae and Roh, 2021; Roh et al., 2018). Three studies had a frequency of two to five times per week without reporting the intensity of the training (Karatrantou et al., 2020; Roh et al., 2020; Violan et al., 1997). It is considered that a greater training frequency may have favored an increase in performance in the 20-m shuttle run test. Likewise, different training intensities can lead to different cardiorespiratory and neuromuscular adaptations (Huerta Ojeda et al., 2017); for example, some training studies that have compared more than one intensity of aerobic exercise while controlling the total volume or energy expenditure of the exercise (Helgerud et al., 2023). Have found significantly more significant increases in aerobic capacity in the higher intensity group (Braith et al., 1994). As indicated above, it is essential to consider how different training intensities can generate different physical and physiological parameters adaptations when selecting an optimal training regimen for a particular sport or population (Gormley et al., 2008).

### 4.3 Jump performance

Meta-analyses of the indirect methods in the standing long jump test showed statistically significant improvements in favor of OCS compared to active/inactive CG. Similar results to those reported by Nam and Lim (2019) in a meta-analysis of Korean non-athlete students showing that taekwondo interventions significantly improved standing broad jumps test by 16.7% (Hedges’s g = 0.431; 95% CI = 0.219 to 0.642, *p* = 0.00) compared to active/inactive CG. Similar to that reported by Cid-Calfucura et al. (2023) in a systematic review of judo, karate, fencing, and boxing athletes who underwent strength training interventions showed improvements (*p* < 0.01) in standing long jump compared to CG. Similar to that reported by Herrera-Valenzuela et al. (2017), young taekwondo athletes who carried out a multicomponent training intervention for 16 weeks showed a significant increase (*p* < 0.05) in standing long jump in males and females. The improvements found in the meta-analyzed studies (Kim et al., 2011; Mohammed, 2020; Sekulic et al., 2006) can be explained by the physical and physiological requirements of the taekwondo and judo sports techniques carried out in the interventions. Taekwondo is an intermittent sport where kicking and punching techniques are used to score points in combat. These actions are executed at high speed, with explosive force predominating (Carroll et al., 2001). Therefore, competitors must be able to move at high speed, with speed and power (Kazemi et al., 2013). Regarding judo, its techniques are characterized by fast and explosive muscular actions; in typical combat, most of the time is spent in grappling disputes (kumi-kata), which require a high level of resistance to the force of upper limbs, while throwing and throwing techniques require high levels of upper and lower limbs muscle strength and power (Franchini et al., 2015). In this sense, given the physical demands of the techniques executed by the students, these may have generated an increase in the lower limbs’ muscle strength through neural adaptations translated into greater recruitment of motor units and fast muscle fibers (Carroll et al., 2001).

Another meta-analysis performed using indirect methods is the Sargent jump test, where no statistically significant improvements were found in favor of OCS compared to active/inactive CG; this is different from the results of Singh et al. (2015), with elite taekwondo athletes who significantly improved (*p* < 0.05) vertical jump after 6 weeks of plyometric training compared to the CG. Similar to the findings reported in Moreno-Azze et al. (2024) in male karatekas, who improved (*p* = 0.01) vertical jump after 6 weeks of plyometric training compared to the CG. The improvements in the standing long jump test compared to the vertical jump test found in the meta-analyzed studies could be explained by the force application vectors of the students’ taekwondo, karate, and judo sports techniques. These techniques are characterized by a predominance of forces in the horizontal vector, with changes in direction and frontal and backward displacements (Ioannides et al., 2020). In this sense, given the movement patterns performed predominantly in a horizontal vector, such as a sequence of forward attacks with kicks and punches or throws and throws in judo, they may have influenced the students to improve the long jump in comparison to the vertical jump given the principle of specificity of strength training (Brearley and Bishop, 2019).

### 4.4 Flexibility

Regarding flexibility, a significant improvement was found in the sit-and-reach test in favor of OCS compared to active/inactive CG. Similar results to those reported by Valdés-Badilla et al. (2021) in a systematic review of older people show increased flexibility through the sit-and-reach test in favor of OCS interventions compared to CG. The same results are presented in adult non-athletes who underwent a boxing intervention for 8 weeks and showed significant improvements (*p* < 0.0001) in the sit-and-reach test compared to an active CG (El Ashker, 2018). In the study by Norambuena et al. (2020) in young judokas who underwent a 5-week suspension training intervention, significant improvements (*p* < 0.01) were reported in the sit-and-reach test compared to traditional judo training. With all of the above, the advances in flexibility in our systematic review may be given by the static stretching performed during the interventions, in conjunction with the repetitive action of techniques such as kicking in taekwondo and throwing techniques in judo, which are similar to dynamic and ballistic type training to increase the range of motion (Mailapalli et al., 2015; Wąsik and Shan, 2014). Dynamic techniques are characterized by having an active movement within the range of motion of the joint required in the sports technique (Degens et al., 2019). In this sense, when participants performed a kicking technique or maximal range of motion in a throwing technique, they performed a dynamic technique to increase flexibility. However, the main difference between dynamic and ballistic OCS techniques is the lack of emphasis on the final phase of the range of motion, and ballistic techniques are performed at the limit of the joint’s range of motion, generating a strong increase in the range of motion (Franchini and Herrera-Valenzuela, 2021). Taking the example of kicking in taekwondo, Wąsik and Shan (2014) mention that during the elevation of the leg in preparation for the kick, the slight bending of the trunk in the direction of the leg that executes the kick is responsible for increasing the range of movement of the hip joint, which would generate a pre-stretch of the hip extensor muscles and contribute to a greater height of the final kick.

### 4.5 Subgroup analysis by type of active control group (CG)

Significant differences were found in the analysis of OCS vs physical education (p < 0.05). However, it is important to mention that only three experimental groups were included *versus* physical education conditions. The OCS used were wrestling and taekwondo, while the activities performed by the physical education group in the taekwondo intervention were not reported (Roh et al., 2018). Improvements in MIHS can be attributed to the sports techniques used in wrestling and taekwondo. For example, wrestling involves constant gripping techniques and isometric actions in the hands and forearms to control the opponent and overcome them through locks with projection techniques in order to obtain a higher score during the fight (Chaabene et al., 2016). Performing these actions repeatedly can result in an improvement in MIHS by increasing the strength of the elbow flexors, which has been significantly correlated with MIHS (Muñoz-Vásquez et al., 2023). Furthermore, the analyzed target groups that performed wrestling followed a strength training program for handgrip through medium resistance therapeutic balls. This may have enhanced the effects of technical training, favoring the significant differences in MIHS compared to the physical education groups (Karatrantou et al., 2020). While the positive effects in the experimental group with taekwondo could be explained by the punches techniques frequently used in attacks and counterattacks (Helgerud et al., 2007), which play a decisive role in the accumulation of points. These actions require rapid force production from the neuromuscular system to transmit the force generated from the ground to the upper limbs and impact the opponent (Ojeda-Aravena et al., 2023). Consequently, during the final phase of the punch, a high isometric muscular tension is generated by clenching the hand, which may be positive for MIHS.

Conversely, no significant differences were found between OCS and recreational activities. However, this may be explained by differences in sample characteristics between the groups analyzed. For example, the taekwondo intervention in the Bae and Roh (2021) was comprised of apparently healthy university students, while the taekwondo intervention by Roh et al. (2020) was comprised of overweight or obese adolescent students. On the other hand, the karate intervention in the Violan et al. (1997) was comprised of healthy adolescent students. Heterogeneity in variables such as age, sex, developmental status, health status, and pre-intervention baseline values in the analyzed studies may have hindered the findings of significant differences in favor of OCS over recreational activities.

### 4.6 Subgroup analysis by age range

Regarding age range, significant differences (p < 0.05) were identified in the sit-and-reach test in favor of OCS in university students compared to active CG. The articles analyzed included taekwondo and judo modalities with a duration of 8 and 16 weeks (Bae and Roh, 2021; Mohammed, 2020). Flexibility is an important quality of physical fitness and a determinant of performance in sports that require the ability to move efficiently through a large range of motion (Donti et al., 2022). As mentioned in the overall meta-analysis for flexibility, the significant improvements in university students may be attributed to the static stretching performed during taekwondo and judo training sessions (Bae and Roh, 2021; Mohammed, 2020). Furthermore, the constant repetition of techniques such as kicking in taekwondo and throwing techniques in judo may have driven the increase in range of motion through increased stretch tolerance and/or decreased tissue resistance to stretch in the sit-and-reach test (Donti et al., 2022; Franchini and Herrera-Valenzuela, 2021).

On the other hand, no significant differences were reported in the sit-and-reach test in favor of OCS in children or adolescents compared to active CG. This is striking because childhood has been suggested to be a key period for developing flexibility (Donti et al., 2022). Specifically, the age range from 6 to 11 years has been proposed as a window of opportunity for flexibility development (Lloyd and Oliver, 2012). This is mainly due to the reduction in musculotendinous stiffness associated with childhood (Donti et al., 2022; Kubo et al., 2001) which may favor greater range of motion and effectiveness of flexibility training (Donti et al., 2022). However, our subgroup analysis did not demonstrate significant increases in the sit-and-reach test in favor of OCS. This could suggest that OCS alone are not sufficient stimulus to improve lower extremity flexibility in children and adolescents, so it may be necessary to include specific flexibility sessions to complement the benefits of OCS. However, our findings should be interpreted with caution given the confounding variables of the analyzed studies, such as training load characteristics, sample sizes used, and differences in health status (e.g., obese children *versus* healthy adolescents).

### 4.7 Subgroup analysis by training dose

Significant differences were found in the sit-and-reach test for <60 min per session in favor of OCS compared to control conditions. No significant improvements were identified for ≥60 min per session in favor of OCS compared to control conditions. This may be attributed to the fact that OCS sessions lasting longer than 60 min cause greater muscle fatigue at both the central and peripheral levels (Tornero-Aguilera et al., 2022). This leads to a decrease in physical performance, as well as the inability of muscles to exert certain levels of muscle tension (Tornero-Aguilera et al., 2022). Consequently, protective inhibitory mechanisms may exist as a result of the activation of the Golgi tendon organ to protect the muscle-tendon unit from damage (Proske and Morgan, 2001). In this context, the muscular tightness produced by training sessions longer than 1 hour could counteract the improvements in the lower limbs in the sit-and-reach test.

### 4.8 Dosage

The dosage used in the OCS interventions ranged from 8 to 39 weeks with a frequency of 1–5 sessions per week of 30–90 min duration at an intensity between 50% and 80% HR_max_. Similarly, Nam and Lim (2019), in a systematic review with meta-analysis, significant improvements (*p* < 0.05) in physical fitness in favor of taekwondo training regarding active/inactive controls in Korean high school athletes with an intervention dose of 12 weeks for 5 weekly sessions of 50 min–60 min with intensities between 50% and 80% HR_max_. Similarly, in the study conducted by Muñoz-Vásquez et al. (2023) in the non-athlete population, significant improvements (*p* = 0.004) in cardiorespiratory fitness were reported in interventions using OCS during 12–36 weeks with 1–5 weekly sessions with a time between 50 min and 120 min with HR_max_ intensities of 50%–86%. Similar to that reported by Valdés-Badilla et al. (2023) in an intervention using taekwondo in older people for 8 weeks with a frequency of three sessions per week with a duration of 60 min at an intensity between 50% and 70% HR_max_ compared to multicomponent training showing significant improvements (*p* < 0.05) in physical fitness in favor of adapted taekwondo. These data allowed us to strengthen our findings in the present meta-analysis. However, it should be understood that physiological responses may vary between athletes and non-athlete population (Degens et al., 2019). In this systematic review, we analyzed individual reports of boxing, karate, and taekwondo in non-athlete students; these OCS involve intermittent actions at different intensities with multidirectional movements performing strikes, grips, and twists (Tabben et al., 2014a). Through this, it was possible to generate adaptations and improvements in various physical fitness assessments of non-athlete students. Combat sports, given their particularities, contemplate different sports techniques with high levels of force applied in short periods, which include everything from actions and movements with punches and kicks mobilizing only one’s body weight to actions with takedowns and grips techniques mobilizing the opponent’s body weight (Chaabene et al., 2018; Cid-Calfucura et al., 2023). These sporting actions depend mainly on explosive strength, maximum strength, and force endurance (Chaabene et al., 2018). In this sense, the practice of combat sports in non-athlete students increased muscle strength through the efforts required during their techniques, developing positive adaptations in the neuromuscular system (Jeknić et al., 2022). Often, in untrained persons, a rapid increase in muscle strength is observed in the early phases of training; these improvements are not only explained by the increase in muscle mass or fiber size, but the increase in strength is due to an adaptation of the neuromuscular system; thus promotes more efficient recruitment of motor units can occur during each effort made (Carroll et al., 2001).

Regarding the certainty of evidence, our systematic review reported it to be moderate to low, which does not allow us to establish definitive recommendations on using OCS to improve physical fitness in non-athlete students by direct and indirect measurements. Similar to that reported by Muñoz-Vásquez et al. (2023) and Valdés-Badilla et al. (2022) in a systematic reviews on the effects of OCS on cardiorespiratory fitness in the non-athlete population and the effects of OCS performed by older people showing a deficient quality of evidence. However, productivity at OCS has increased in recent years. Different systematic reviews have been published on strength training (Cid-Calfucura et al., 2023) and plyometrics (Ojeda-Aravena et al., 2023) in OCS, HIIT (Vasconcelos et al., 2020) in OCS, and nutritional aspects and ergogenic (Vicente-Salar et al., 2022) in OCS. Despite this, few systematic reviews have explored the effects of OCS in non-athlete populations of different ages (Valdés-Badilla et al., 2022), which enhances and highlights this systematic review.

### 4.9 Strengths and limitations

As limitations we find: (i) moderate to high heterogeneity in the meta-analysis performed, probably due to variations in study designs, participant characteristics (age, sex, health status), and intervention protocols; (ii) the lack of reporting of the intensity of the activities performed (only four studies mentioned it), which makes replication of these interventions difficult; (iii) the lack of studies that used boxing as an intervention, which reduces the generalizability of our findings; and (iv) not analyzing reactions to OCS interventions that are psychophysiological, physiological, and/or biochemical, and how these effects change with age in non-athlete students also how they may impact body posture (Domaradzki et al., 2021), as well as on central nervous system function (Li Y. et al., 2022), responses on mental health, cognitive function (Ciaccioni et al., 2024) and academic achievement (Pinto-Escalona et al., 2021) how these effects vary with age in non-athlete students. In the strengths, we found: (i) the methodological quality above 60% in the studies analyzed; (ii) the methodological processes that followed the PRISMA, PROSPERO, TESTEX, RoB 2 and GRADE scales; (iii) the use of seven databases: PubMed, EBSCOhost, Medline, Scopus and Web of Science (core collection) and; (iv) analysis of the effect of OCS in non-athlete students. The results presented in this systematic review hold substantial potential, demonstrating that OCS interventions may provide unique motivational benefits, particularly for student populations. The dynamic, skill-based, and competitive nature of OCS can enhance intrinsic motivation, engagement, and adherence to physical activity, especially among youth who may not respond positively to traditional physical education formats (Lakes and Hoyt, 2004). Moreover, OCS training integrates components that support the long-term development of physical literacy, including movement competence, physical capacity, and conceptual understanding of physical activity, while simultaneously promoting emotional regulation through the cultivation of discipline, self-control, and resilience developed in training and sparring contexts (Lakes and Hoyt, 2004). However, given that OCS is an alternative to enhance health (Valdés-Badilla et al., 2021) and exhibits a strong desire and adherence to the practice of these sports, further support and research are required (Muñoz-Vásquez et al., 2023; Silva and Quaresma, 2019; Valdés-Badilla et al., 2021). To conduct new systematic reviews that could address other aspects of health status like psychophysiological, physiological, and/or biochemical responses, more studies with high-quality methodology (e.g., double-blind randomization, supervised control groups, and previously registered research protocols) and further description of physical exercise (technical rationale) and intensity of the activities performed are required (Morales et al., 2020), along with studies on psychophysiological, physiological, and/or biochemical reactions to OCS interventions, how these effects change with age in students who are not athletes and also how they may impact postural control (Domaradzki et al., 2021), as well as central nervous system function (Li Y. et al., 2022), and additionally, responses to mental health, cognitive function (Ciaccioni et al., 2024) and academic achievement (Pinto-Escalona et al., 2021), and how these effects vary with age in non-athlete students.

## 5 Conclusion

OCS improves standing long jump as well as lower body flexibility. It does not show improvements in cardiorespiratory fitness, upper body muscle strength and vertical jump height. However, with respect to dosage and age range <60 min per session in university students is adequate to improve lower body flexibility. OCS is more effective in improving upper body muscle strength compared to physical education.

## Data Availability

The datasets presented in this study can be found in online repositories. The names of the repository/repositories and accession number(s) can be found in the article/Supplementary Material.
