# Supplementary material for: Effects of olympic combat sports on physical fitness in non-athlete students: a systematic review with meta-analysis
Source: Front Physiol. 2025 Jul 14;16:1620621. doi: 10.3389/fphys.2025.1620621 (PMC12301306; doi:10.3389/fphys.2025.1620621)
Supplement: Supplementary file 1 [file DataSheet1.pdf]

Supplementary Figures

Group by Subgroup within study	Study name	Subgroup within study	Hedges's g	Standard error	Variance	Lower limit	Upper limit	Z-Value	p-Value
P.E. vs OCS	Karatrantou et al. (2020) A	P.E. vs OCS	2,264	0,421	0,177	1,438	3,089	5,375	0,000
P.E. vs OCS	Karatrantou et al. (2020) B	P.E. vs OCS	1,328	0,362	0,131	0,619	2,036	3,672	0,000
P.E. vs OCS	Roh et al. (2018)	P.E. vs OCS	0,219	0,356	0,127	-0,480	0,917	0,614	0,539
P.E. vs OCS			1,254	0,580	0,336	0,118	2,390	2,163	0,031
R.A vs OCS	Bae et al. (2021)	R.A vs OCS	0,143	0,395	0,156	-0,631	0,916	0,362	0,718
R.A vs OCS	Roh et al. (2020)	R.A vs OCS	0,038	0,428	0,183	-0,802	0,877	0,088	0,930
R.A vs OCS	Violan et al. (1997)	R.A vs OCS	-0,344	0,403	0,162	-1,133	0,446	-0,853	0,394
R.A vs OCS			-0,055	0,235	0,055	-0,517	0,406	-0,235	0,814

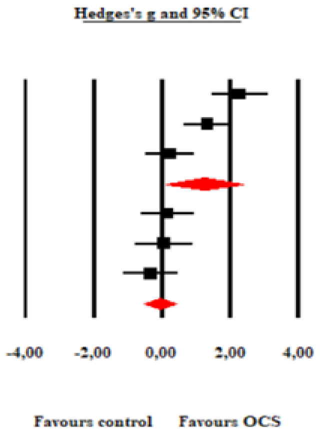

Figure 9. Forest plot of changes in performance on the MIHS in students practicing Olympic combat sports compared to an active control group, stratified by type of control group activities. The values shown correspond to effect sizes (Hedges' g) with 95% confidence intervals (CI). The squares represent the effect sizes of each study, with their size indicating the statistical weight of each study within the meta-analysis. The studies are divided into two subgroups: physical education (P.E.) vs. Olympic combat sports (OCS) and recreational activity (R.A.) vs. Olympic combat sports (OCS). Positive values favor students practicing Olympic combat sports, while negative values favor the active control group. The red diamonds represent the pooled effect sizes for each subgroup.

Study name	Subgroup within study	Hedges's g	Standard error	Variance	Lower limit	Upper limit	Z-Value	p-Value
Kim et al. (2011)	Children and/or adolescents	0,668	0,384	0,147	-0,084	1,420	1,740	0,082
Roh et al. (2018)	Children and/or adolescents	-0,023	0,355	0,126	-0,720	0,673	-0,065	0,948
Roh et al. (2020)	Children and/or adolescents	0,382	0,433	0,187	-0,466	1,229	0,882	0,378
Sekulic et al. (2006)	Children and/or adolescents	1,677	0,236	0,056	1,215	2,139	7,110	0,000
		0,707	0,434	0,188	-0,143	1,558	1,629	0,103
Bae et al. (2021)	University students	0,572	0,403	0,162	-0,218	1,361	1,419	0,156
Mohammed et al. (2020) A	University students	1,372	0,287	0,082	0,809	1,935	4,778	0,000
Mohammed etl al. (2020) B	University students	0,684	0,261	0,068	0,173	1,195	2,626	0,009
		0,903	0,255	0,065	0,404	1,403	3,546	0,000

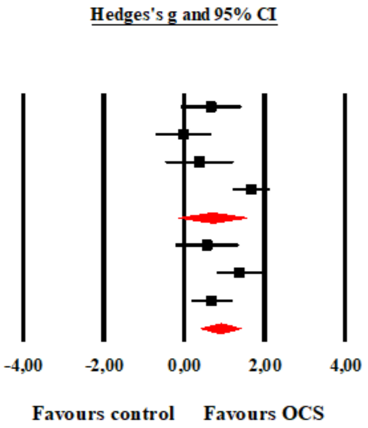

Figure 10. Forest plot of changes in performance on the sit-and-reach test in students practicing Olympic combat sports compared to an active control group, stratified by age group. The values shown correspond to effect sizes (Hedges' g) with 95% confidence intervals (CI). The squares represent the effect sizes of each study, with their size indicating the statistical weight of each study within the meta-analysis. The studies are divided into two subgroups: children and/or adolescents, and university students. Positive values favor students practicing Olympic combat sports, while negative values favor the active control group. The red diamonds represent the pooled effect sizes for each subgroup.

Study name	Subgroup within study	Hedges's g	Standard error	Variance	Lower limit	Upper limit	Z-Value	p-Value
Kim et al. (2011)	<60 min	0,668	0,384	0,147	-0,084	1,420	1,740	0,082
Mohammed et al. (2020) A	<60 min	1,372	0,287	0,082	0,809	1,935	4,778	0,000
Mohammed etl al. (2020) B	<60 min	0,684	0,261	0,068	0,173	1,195	2,626	0,009
Sekulic et al. (2006)	<60 min	1,677	0,236	0,056	1,215	2,139	7,110	0,000
		1,131	0,264	0,070	0,613	1,648	4,280	0,000
Bae et al. (2021)	60 min or more	0,572	0,403	0,162	-0,218	1,361	1,419	0,156
Roh et al. (2018)	60 min or more	-0,023	0,355	0,126	-0,720	0,673	-0,065	0,948
Roh et al. (2020)	60 min or more	0,382	0,433	0,187	-0,466	1,229	0,882	0,378
		0,277	0,227	0,051	-0,168	0,721	1,221	0,222

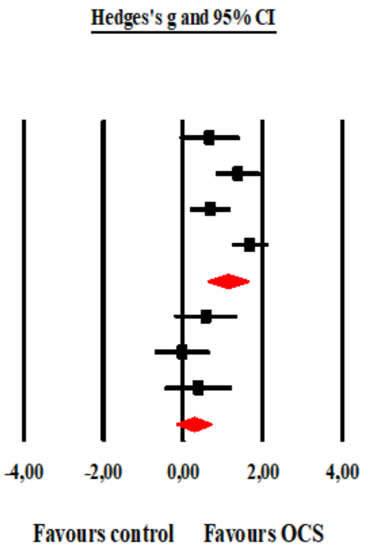

Figure 11. Forest plot of changes in performance on sit-and-reach test in students practicing Olympic combat sports compared to an active control group, stratified by session duration. The values shown correspond to effect sizes (Hedges' g) with 95% confidence intervals (CI). The squares represent the effect sizes of each study, with their size indicating the statistical weight of each study within the meta-analysis. The studies are divided into two subgroups: sessions lasting less than 60 minutes and sessions lasting 60 minutes or more. Positive values favor students practicing Olympic combat sports, while negative values favor the active control group. The red diamonds represent the pooled effect sizes for each subgroup.
